# Assessment of Haemostasis in patients undergoing emergent neurosurgery by rotational Elastometry and standard coagulation tests: a prospective observational study

**DOI:** 10.1186/s12871-017-0440-1

**Published:** 2017-10-24

**Authors:** Christoph Ellenberger, Najia Garofano, Gleicy Barcelos, John Diaper, Gordana Pavlovic, Marc Licker

**Affiliations:** 1 Department of Anesthesiology, Pharmacology and Intensive Care, University Hospital of Geneva, -1211 Geneva, CH Switzerland; 2 Faculty of Medicine, University of Geneva, -1211 Geneva, CH Switzerland

**Keywords:** Brain injury, Trauma, Transfusion, Anesthesia, Bleeding

## Abstract

**Background:**

Rotational elastometry (ROTEM) has been shown useful to monitor coagulation in trauma patients and in major elective surgery. In this study, we aimed to evaluate the utility of ROTEM to identify hemostatic disturbances and to predict the need for transfusion, compared with standard coagulation tests (SCTs) in patients undergoing emergent neurosurgery.

**Methods:**

Over a four-year period, adult patients who met criteria for emergent neurosurgery lasting more than 90 min were included in the study. Blood was collected preoperatively and analyzed with SCTs (international normalized ratio [INR], fibrinogen concentration, prothrombin time [PT or Quick], partial thromboplastine time [PTT], fibrinogen concentration and platelet count), and ROTEM assays. Correlations between SCTs and ROTEM parameters as well as receiver operating characteristic curves were performed to detect a coagulopathic pattern based on standard criteria and the need for transfusing at least 3 units of packed red blood cells (PRBCs).

**Results:**

In a cohort of 92 patients, 39 (42%) required ≥3 PRBCs and a coagulopathic pattern was identified in 32 patients based on SCTs and in 19 based on ROTEM. There was a strong correlation between PTT and INTEM coagulation time (*R* = 0.76) as well as between fibrinogen concentrations and FIBTEM maximal clot firmess (*R* = 0.70). The need for transfusion (≥ 3 PRBCs) was best predicted by the maximal clot firmess of EXTEM and FIBTEM (AUC of 0.72 and 0.71, respectively) and by fibrinogen concentration (AUC of 0.70).

**Conclusions:**

In patients undergoing emergent neurosurgery, ROTEM analysis provides valid markers of early coagulopathy and predictors of blood transfusion requirements.

## Background

Surgical complications increase the cost of health care worldwide and directly contribute to patient morbidity and mortality [1]. After cranial or spinal interventions, the most frequent postoperative complication is bleeding requiring blood transfusion, followed by re-intervention and failure to wean from the ventilator [2]. In the particular settings of brain injury following trauma or neurosurgical procedures, abnormal patterns of coagulation may develop. Depending on different diagnostic criteria, the incidence of acute traumatic coagulopathy varies between 7% and 86% after isolated traumatic brain injury [3] and it is associated with single-and multiple organ dysfunction, greater transfusion requirements, higher mortality and, prolonged stay in the intensive care unit (ICU) [4].

According to the current hypothesis, trauma brain injuries are associated with a combination of hyper- and hypocoagulable states. The initial brain damage triggers the release of tissue factor and initiate the coagulation process which is further amplified and sustained by secondary brain ischemic and hemorrhagic injuries [5]. Concomittantly, hypoperfusion and endothelium damage result in thrombomodulin release with stimulation of protein C pathway leading to the inhibition of activated co-factors V and VIII and decreased thrombin generation leading to a decrease in fibrin formation [6]. Likewise, intracranial hypertension and brain ischemia in the context of brain hematoma/tumor, have also been shown to initiate the coagulation cascade in non-trauma patients [6, 7]. Variable tissue release of thrombin and plasminogen activator, along with activation of the protein C pathway and platelet dysfunction may all concur to these acute hemostatic disorders. Moreover, the efficacy of antifibrinolytic agents in reducing blood losses in patients with brain trauma, subarachnoid hemorrhage or intracranial tumor emphasizes the importance of hyperfibrinolysis in patients undergoing major neurosurgery [8–10].

Standard coagulation tests (SCTs) including the platelet count, plasma fibrinogen levels, the international normalized ratio (INR), partial thromboplastin time (PTT) and prothrombin time (PT or Quick test) are the mainstay in the anesthesiologist’s armoury to assess preoperatively hemostatic disturbances and to predict the need transfusion [11]. More recently, viscoelastic hemostatic assays (VHA) such as thromboelastography (TEG) and rotational elastometry (ROTEM) have emerged as suitable monitors of the coagulation process during the perioperative period [12, 13]. These VHA Point-Of-Care (POC) devices both measure and display the viscoelastic properties in whole blood samples from the initial phase of fibrin formation to clot retraction and fibrinolysis. In contrast with SCTs performed in plasma samples and in remote laboratory settings, these POC-VHA deliver bedside information on the dynamic interactions between plasmatic factors and circulating blood cells. In cardiac surgery, the implementation of POC-VHA has been associated with a reduction in blood transfusion requirements [14]. So far, no study has been conducted on the utility of POC-VHA in patients undergoing emergent neurosurgery [15].

The purpose of this observational study was to compare preoperative SCTs to ROTEM-derived parameters in patients scheduled for emergent neurosurgical interventions. We hypothesized that the use of ROTEM may identify specific coagulation abnormalities and could be considered as a suitable alternative for SCTs.

## Methods

### Patient population

From January 2011 to December 2014, adult patients (>18 years) undergoing emergent neurosurgical procedures at the University Hospital of Geneva were included in this prospective trial if they fulfilled the following criteria: 1) intracranial intervention, 2) necessity to proceed to surgery within 6–8 h after diagnostic work up, 3) surgery lasting at least 90 min. The exclusion criteria were a life expectancy <24 h and/or any associated pathology requiring a combined surgical procedure (e.g., multiple trauma). The indications for surgical operations were: severe traumatic brain injury, space-occupying intracerebral hematoma or expanding/ruptured aneurysm, tumoral process associated with raising intracranial pressure or neurological deficit. A severe isolated traumatic brain injury was defined by an Abbreviated Injury Scale for head (AIS_head_) > 3, with an AIS for extracranial injuries (AIS_extracranial_) < 3, where 3 is rated as “serious [16].

The study was approved by the local institutional research ethics board (CCER N°10–128) and informed consent was waived given the emergency context and the fact that ROTEM testing was routinely performed in all surgical patients at high risk of bleeding.

### Study design

**Fig. 1:**
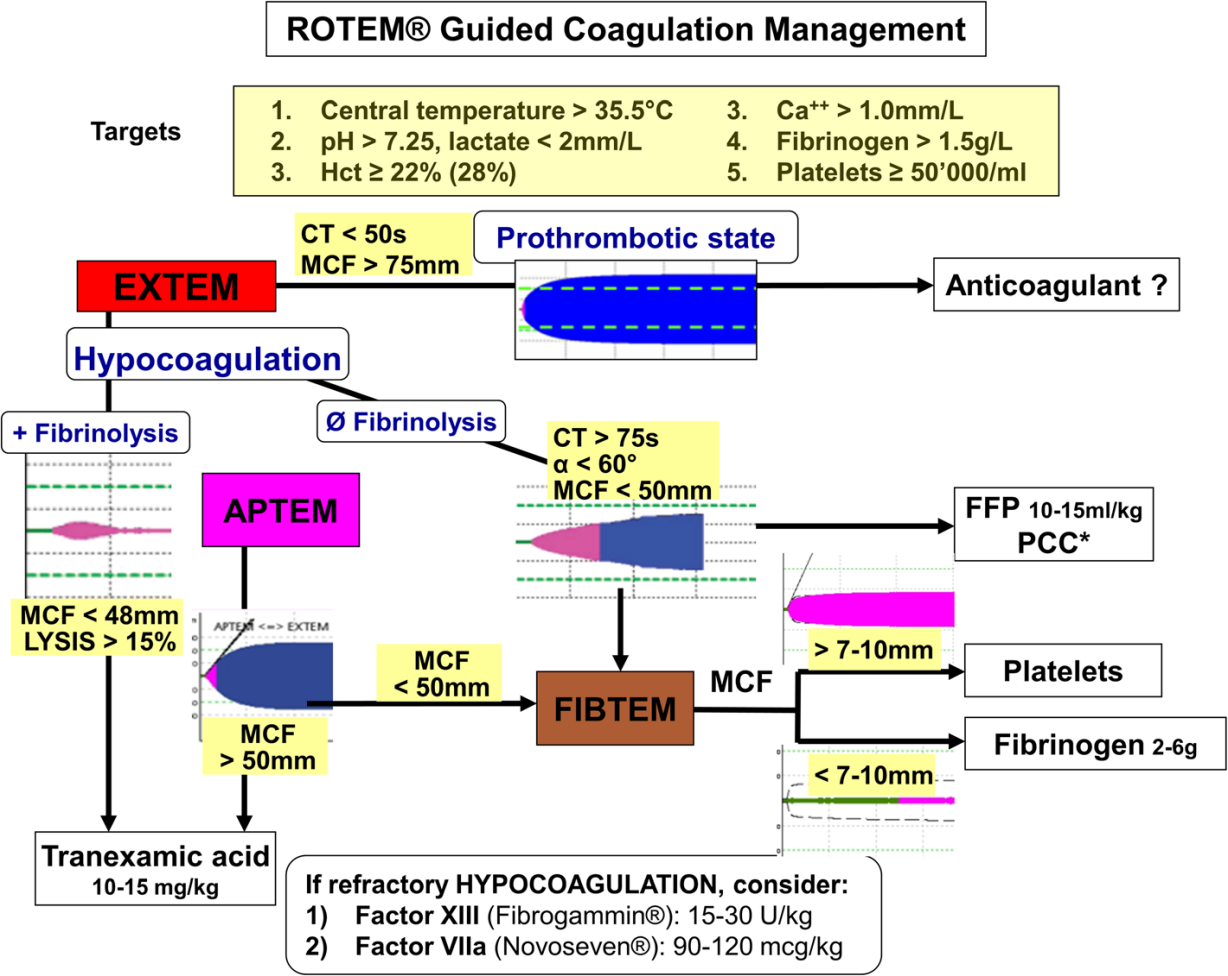
Goal-Directed Treatment of hemostatic disturbances based on ROTEM analysis**.** *Prothrombin Complex Concentrate

This study was performed as a prospective unblinded observational investigation. Intraoperatively, the hemostatic therapy (fresh frozen plasma [FFP], prothrombin complex concentrate [PCC], platelet concentrates, activated factor VII) was guided by the results ROTEM testing as well as by clinical judgement (Fig. 1). This algorithm had been implemented 2 years before the start of the study in our department, for all types of major surgery [17].

### Patient management

Anesthesia was induced and maintained intravenously with propofol, sufentanil and rocuronium or atracurium. After tracheal intubation, mechanical ventilation was delivered with a low tidal volume (6–8 ml/kg of ideal body weight) and a low positive end-expiratory pressure (4–8 cmH_2_O) while the respiratory frequency was adjusted to maintain normocapnia (end-expiratory carbon dioxide pressure between 38 and 42 mmHg). All patients were equipped with two peripheral venous catheters, an invasive radial arterial line, a nasopharyngeal temperature probe and a bladder catheter. Intraoperatively, body temperature was maintained by forced-air warming (Bair Hugger™ 3 M, St. Paul, USA) and by warming intravenous fluids. Pneumatic compression boots were placed after anesthesia induction to prevent deep vein thrombosis.

A standardized fluid management approach guided by invasive hemodynamic parameters was aimed to maintain euvolemia with the infusion of isotonic fluids administration (2–3 ml/kg during anesthesia induction followed by a continuous rate of 1.5–2.5 ml/kg/h and additional fluid volume if necessary). Blood losses were substituted in a 2:1 ratio with a balanced crystalloid solution (Ringer-acetate; B. Braun, AB). One or two units of PRBC were administered if the hemoglobin concentration decreased below 90 g/L.

### Coagulation testing

Venous blood samples for SCTs were collected preoperatively in 5 mL citrated tubes in the emergency department. The SCTs were performed in the settings of the Central Laboratory and included the followings: PTT, PT (Quick), INR, and plasma fibrinogen concentration (Clauss method; Siemens-Dade Behring Healthcare Diagnostics, Marburg, Germany). Platelet count, hemoglobin concentration and hematocrit were measured in EDTA blood samples with a Coulter Counter (Beckman Coulter Diagnostics, Brea, CA, USA).

For ROTEM analysis, blood was sampled in the operating theater on citrated tubes from the radial arterial catheter with a continuous heparin-free sodium chloride flushing system. ROTEM analyses were performed within minutes of blood sampling by anesthesia nurses or physicians trained to perform the ROTEM tests according to the manufacturer’s instructions (ROTEM®; TEM Innovations GmbH, Germany) [18]. Four ROTEM analyses were performed in parallel: an extrinsically activated assay with recombinant tissue factor (EXTEM), an intrinsically activated test using phospholipid–ellagic acid (INTEM), a platelet inhibition test using Cytocholasin D (FIBTEM) and an aprotinin-based test to detect ongoing hyperfibrinolysis (APTEM). The following ROTEM variables were measured: clotting time (CT [sec], the time from start of measurement until formation of a clot with an amplitude of 2 mm); clot formation time (CFT [sec], time from the end of CT [amplitude of 2 mm] until a clot firmness of 20 mm is achieved); alpha angle, angle between the centre line and a tangent to the curve through the 2 mm amplitude point; maximum clot firmness (MCF [mm], the final strength of the clot, resulting from the interaction of fibrin, activated platelets and factor XIII) and, clot amplitude after 15 min (CA15 [mm], amplitude of clot firmness after 15 min).

### Measurements and study endpoints

Using SCTs, coagulopathy was defined as one or more of the following results: Quick (PT) < 70%, INR > 1.3, aPTT >35 s, fibrinogen <1.5 g/L, and platelet count <100′000 /mcL [18]. Using ROTEM parameters, coagulopathy was defined with two or more of the following results: EXTEM CT > 80 s, EXTEM CFT > 159 s, EXTEM MCF < 50 mm, INTEM CT > 240 s, INTEM CFT > 110 s, INTEM MCF < 50 mm, and FIBTEM MCF < 9 mm [18–21].

Demographic, clinical and surgical data, Glasgow Coma Scale (GCS) score upon hospital admission, administration of fluids, blood transfusion products (FFPs, PCC, thrombopheresis) and other hemostatic agents (recombinant factor VII [rFVII], tranexamic acid [TA]) were all recorded from the anesthesia, surgical and emergency charts. On the morning of the first day following surgery, venous blood was collected to perform SCTs and ROTEM analysis. In addition, the electronic medical files were examined to report hospital and ICU length of stay as well as major postoperative complications (in-hospital mortality, acute respiratory failure, infections, renal failure, thromboembolic events, myocardial infarct, and heart failure).

### Statistical analysis

In our institutional database, we found that 85% of patients undergoing emergent neurosurgical procedures were transfused, the median PRBC transfusion was 2 units and 42% of patients received at least 3 RBCs. Hence, the cohort of patients was divided into two groups, high bleeders (HB) receiving at least 3 PRBCs or requiring re-operation for hematoma drainage and, low bleeders (LB) receiving less than 3 PRBCs and not requiring re-operation for hemostasis. Summary descriptive statistics are expressed as frequencies (and percentages, %), medians (and interquartile range, IQ25–75%), or means (and standard deviations, SD). Clinical data and coagulation parameters as well as postoperative outcome were compared between the two groups (HB and LB) with the chi-squared test for categorical variables and with the two-sided unpaired Student t test (normal distribution) or Wilcoxon rank test (non-normal distribution) for continuous variables.

The Pearson product-moment correlation coefficient was used to assess the agreement between SCTs and ROTEM parameters that mostly follow a normal distribution and measure similar aspects of the coagulation cascade (i.e. PT, aPTT and INR with CT; fibrinogen and platelet with alpha angle and MCF).

The area under the curve (AUC) of the receiver operating characteristic (ROC) curves was determined for variables that were significantly related with PRBC transfusion. The ROC analysis evaluates diagnostic/predictive accuracy by changing the laboratory cut-off point throughout the potential range of the test under study to examine the specificity and sensitivity of the tests [22]. All analyses were performed using STATA 14 software (Stata Corp, College Station, TX, USA) and statistical significance was specified as a two-tailed type I error (*P* value) set below the 0.05 level.

## Results

During a 3-year period, 112 patients undergoing emergent neurosurgery were consecutively enrolled in this prospective study. Twenty patients were excluded due to minor or short lasting interventions (minor procedures, *N* = 5; surgery lasting less than 90 min, *N* = 15). In total, data from 92 patients were analyzed, their mean age was 52 years (SD 17.7 yrs), 49 (53%) patients were male and 29 (32%) were admitted to the hospital with severe trauma brain injury. The median duration of the neurosurgical procedures was 180 min (IQ25–75, 113–249 min) and in-hospital mortality was 27.1%.

No patient required re-operation for hematoma drainage and 39 (42%) required three or more PRBCs (HB group). As shown in Table 1, the HB group (*N* = 39) was characterized by a higher proportion of trauma patients, lower Glasgow Coma Score, higher heart rate and lower hemoglobin level, compared with the LB group (*N* = 53).

**Table 1:** Demographic and clinical characteristics of patients undergoing emergency neurosurgery

Characteristics	High Bleeders	Low Bleeders	*P*-value
	(*N* = 39)	(N = 53)	
Demographic data			
Age, years^a^	51.3 (18.5)	52.5 (17.3)	0.746^c^
Weight, kg^a^	71.4 (13.9)	75.4 (15.2)	0.204^c^
Height, cm^a^	170.7 (9.5)	170.5 (9.9)	0.925^c^
Body Mass index, kg/(m^2^)^2a^	24.4 (3.0)	25.7 (4.3)	0.146^c^
Sex, male	22 (56.4)	27 (50.9)	0.604
Glasgow Coma Score^b^	5 (3–7)	10 (5.5–13)	0.008^d^
ASA 3 & 4	33 (84.6)	38 (71.7)	0.145
Admission for Trauma	19 (48.7)	10 (18.9)	0.005
Comorbidities			
Arterial hypertension	9 (23.1)	16 (30.2)	0.449
Chronic kidney disease	1 (2.6)	1 (1.9)	1.000^e^
Diabetes mellitus	2 (5.1)	9 (17.0)	0.110^e^
Cardiac arrhythmia	1 (2.6)	5 (9.4)	0.237^e^
Preoperative medications			
Antiplatelet	8 (20.5)	10 (18.9)	0.844
Anti-vitamine K	3 (7.7)	2 (3.8)	0.647^e^
Thrombine-inhibitor	5 (12.8)	7 (13.2)	0.957
Hemodynamics upon arrival in the operating theater			
Systolic arterial pressure^a^, mmHg	132 (34.0)	140 (29.4)	0.231^c^
Heart rate^a^, beat/min	89 (22)	80 (19)	0.026^c^
Laboratory tests			
Hemoglobin, g/dl^a^	10.7 (2.1)	11.6 (2.1)	0.047^c^
Lactate^b^, mm/L	1.4 (0.8–2.2)	1.0 (0.8–1.7)	0.115^d^

*ASA* American Society Association physical status classification, *TRISS* Trauma and Injury Severity Score

Data given as number (percentage) unless otherwise indicated;

^a^data given as mean (standard deviation)

^b^data given as median (range)

Chi-squared tests were used for statistical tests unless otherwise indicated;

^c^student t test

^d^Wilcoxon rank-sum test

^e^Fisher exact test

Before surgery, a coagulopathic pattern was identified in 32 patients (34.8%) based on SCTs, 19 (48.7%) in the HB group and 13 (24.5%) in the LB group. Based on ROTEM analysis a coagulopathic pattern was identified in 19 patients (20.6%), 15 (38.5%) in the HB group and 4 (7.6%) in the LB group. Agreement between SCTs and ROTEM analysis in detecting (or not) a coagulopathic pattern was achieved in 61 patients (66.3%).

Preoperatively, there was a strong correlation between PTT and both CT and alpha angle of INTEM (*R* = 0.76 and 0.73, respectively) and between fibrinogen plasma concentrations and the MCF of FIBTEM (*R* = 0.70). There was a moderate correlation between preoperative platelet count and the MCF of EXTEM (*R* = 0.61). The correlation between preoperative PT/INR and the CT and alpha angle of EXTEM was weak (< 0.60).

Regarding preoperative coagulation testing (Table 2), patients in the HB group presented significantly lower plasma fibrinogen concentrations, lower Quick values and higher INR whereas ROTEM analysis demonstrated significantly prolonged CT and CFT at the EXTEM assay as well as lower MCF and CA15 at the EXTEM, INTEM, FIBTEM and APTEM assays, compared with the LB group. As shown in Table 3, surgical indications were similar in the two groups. In the HB group surgical time was longer and patients were more likely to be treated with FFPs, fibrinogen, TA and PCC than the LBs.

**Table 2:** Preoperative coagulation parameters of patients undergoing emergent neurosurgery

	High Bleeders	Low Bleeders	*P*-value
	(N = 39)	(*N* = 53)	
Standard coagulation tests			
Platelets, 10^3^/mcL^a^	178 (87)	194 (86)	0.403^c^
Quick, %	82 (62–94)	94 (79–100)	0.018
INR, a.u.^a^	1.22 (0.30)	1.07 (0.08)	0.005^c^
Partial Thromboplastine Time, sec	34 (27–38)	30 (26–32)	0.383
Fibrinogen, g/L^a^	2.5 (1.2)	3.1 (0.9)	0.006^c^
Coagulopathy, (%)^b^	19 (61.3)	13 (33.3)	0.032^d^
Thromboelastometry			
EXTEM			
CT (s)	65 (50–88)	52 (47–62)	0.002
CFT (s)	136 (91–217)	104 (74–129)	0.001
alpha Angle	70 (60–78)	73.5 (69.5–78)	0.129
MCF (mm)	54.5 (45–63)	64 (57–69)	<0.001
INTEM			
CT (s)	157 (133–180)	154 (141–167)	0.681
CFT (s)	88 (68–134)	69 (57–97)	0.005
alpha Angle	73 (64–79)	77 (74–79)	0.037
MCF (mm)	57 (50–63)	65 (57–70)	0.001
FIBTEM			
MCF (mm)	12 (7–16)	16 (14–22)	<0.001
Coagulopathy, (%)^b^	15 (45.6)	4 (9.3)	<0.001^d^

*a.u.* arbitrary unit, *CT* coagulation time, *CFT* clot formation time, *INR* international normalized ratio, *MCF* maximal clot firmness

Data given as median (range) unless otherwise indicated;

^a^data given as mean (standard deviation)

^b^data given as number (percentage)

Wilcoxon rank-sum test was used for statistical tests unless otherwise indicated;

^c^student t test

^d^Chi-squared tests

**Table 3:** Surgical characteristics and intraoperative fluid and hemostatic management

	High Bleeders	Low Bleeders	*P*-value
	(N = 39)	(N = 53)	
Surgical features			
Decompressive craniectomy	10 (25.6)	14 (26.4)	0.933
Subdural hematoma	11 (28.2)	14 (26.4)	0.849
Subarachnoid haemorrhage	12 (30.8)	13 (24.5)	0.469
Subdural hematoma & subarachnoid haemorrhage	6 (15.4)	7 (13.2)	0.767
Surgery time, (min)^c^	190 (159–280)	156 (90–230)	0.012^d^
Intraoperative Fluids			
Crystalloids, ml/min^a^	21.8 (10.4)	18.5 (16.4)	0.272^b^
Colloids, ml/min^c^	0 (0–2.8)	0 (0–0.8)	0.312^d^
Blood products & haemostatic therapy			
Blood transfusion	39 (100)	28 (52.8)	<0.001
Units/patient transfused^c^	5 (3–7)	1 (1–2)	<0.001^d^
Fresh frozen plasma	27 (69.2)	12 (22.6)	<0.001
Units/patient transfused^c^	3 (2–7)	2 (1–2.5)	0.002^d^
Platelets	11 (28.2)	9 (17.0)	0.197
Units/patient transfused^c^	2 (1–3)	2 (1–3)	0.812^d^
Prothrombin Complex Concentrate	7 (18.0)	2 (3.8)	0.033^e^
10^3^ units/patient treated^c^	1.8 (1.2–2.4)	3 (2.4–3.6)	0.133^d^
Fibrinogen	29 (74.4)	11 (20.8)	<0.001
g/patient treated^c^	4 (2–6)	4 (2–4)	0.205^d^
Recombinant Factor VII	3 (7.7)	0 (0.0)	0.073^e^
mg/patient treated^c^	7 (5–9)	0 (0–0)	NA
Tranexamic acid	7 (18.0)	1 (1.9)	0.009^e^
g/patient treated^c^	1.5 (1.0–2.0)	1.0 (1.0–1.0)	0.368^d^

Data given as number (percentage) unless otherwise indicated. Chi-squared tests were used for statistical tests unless otherwise indicated;

^a^Data given as mean (standard deviation)

^b^student t test

^c^data given as median (range)

^d^Wilcoxon rank-sum test

^e^Fisher exact test

**Fig. 2:**
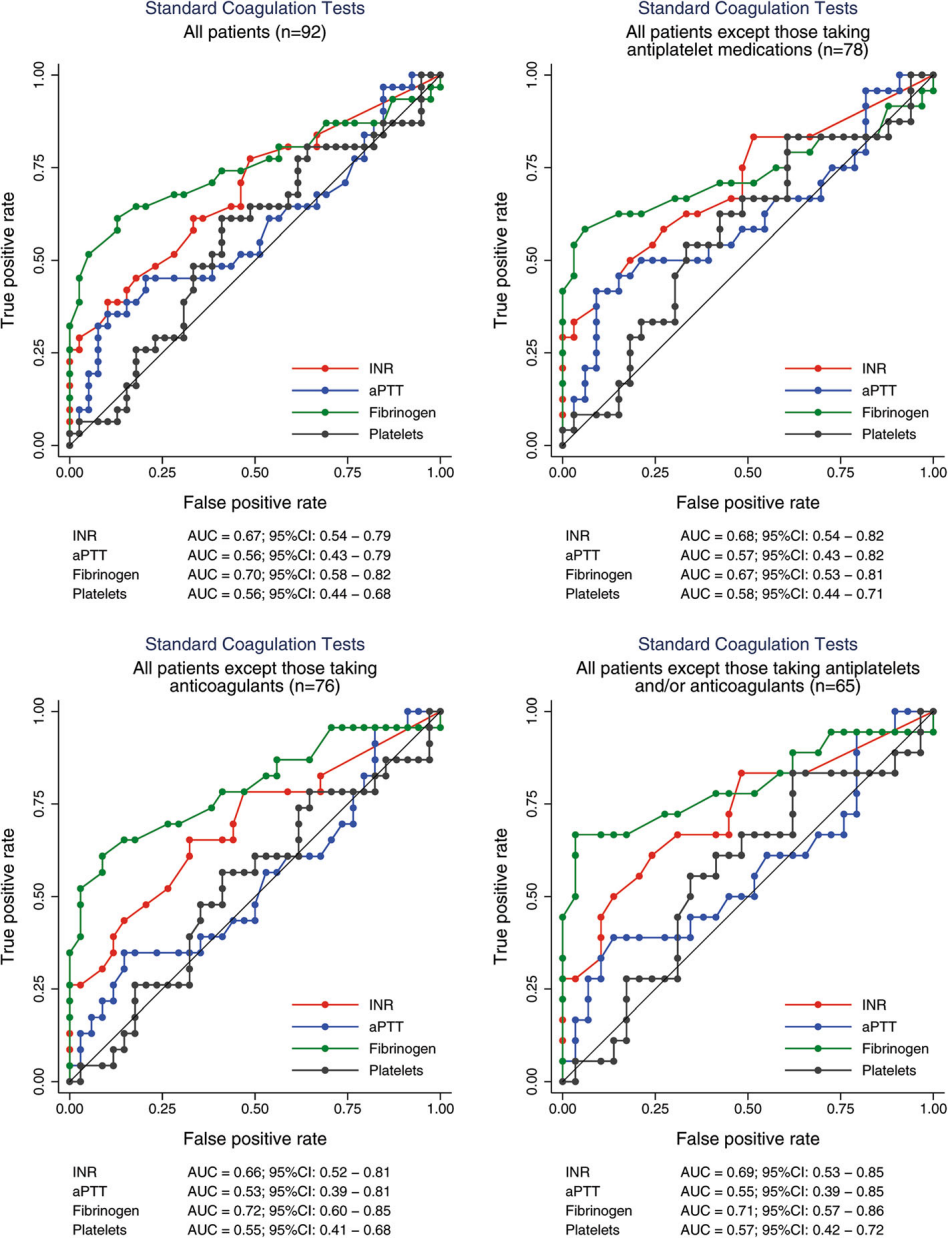
Receiver operating characteristic (ROC) analysis of standard coagulation tests to predict transfusion of at least 3 packed red blood cell units

**Fig. 3:**
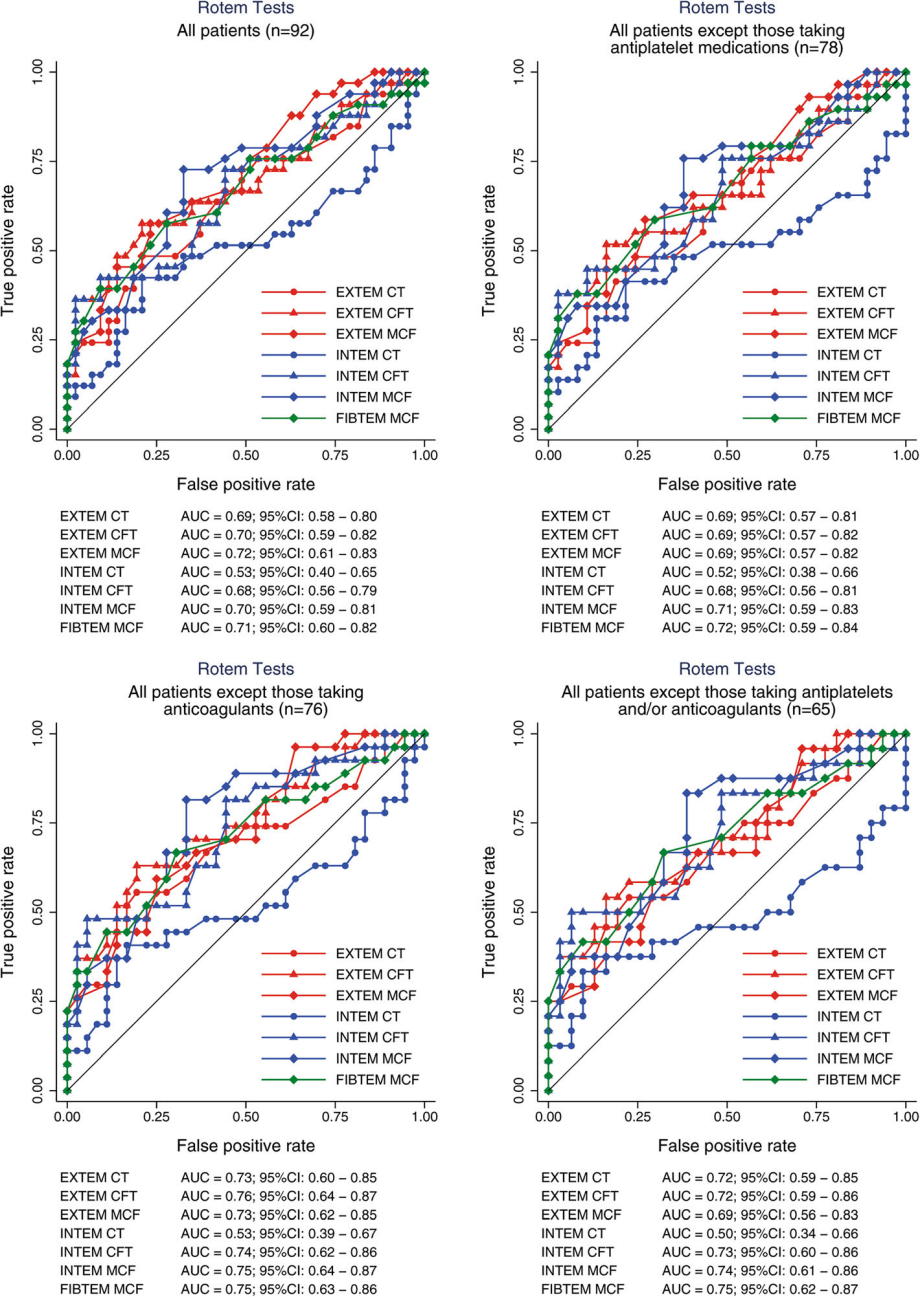
Receiver operating characteristic (ROC) analysis of rotational elastometry (Rotem) to predict transfusion of at least 3 packed red blood cell units

Transfusion with at least 3 PRBCs was best predicted by the Glasgow Coma Score (AUC of 0.74, and 95%CI between 0.58–0.89). As shown in figs. 2 and 3, the AUC of the ROC analysis for identifying patients receiving at least 3 PRBCs was 0.72 for MCF-EXTEM (95%CI 0.61–0.83), 0.71 for MCF-FIBTEM (95%CI 0.60–0.82), 0.70 for INTEM-MCF (95%CI 0.59–0.81) and, 0.70 for fibrinogen levels (95%CI 0.58–0.82). Removing patients treated with anticoagulant and/or antiplatelet drug did not significantly alter the main ROC results, the largest AUC were still achieved for fibrinogen measurements (0.71, 95%CI 0.57–0.86), INTEM-MCF (0.74, 95%CI 0.61–0.86) and MCF-FIBTEM (0.75, 95%CI 0.62–0.87). The accuracy in predicting transfusion ≥3 PRBCs, was equal or superior with ROTEM-derived parameters compared with SCTs (Table 4). Indeed, 64.1% patients were correctly classified as HB (or LB) based on SCTs, whereas the accuracy for correct classification was 69.6% when using ROTEM based parameters. To predict the need for transfusion (≥ 3 PRBCs), ROTEM analysis was more sensitive than SCTs (78.9% versus 59.4), whereas specifities were similar (67.1% and 66.7% respectively). The best thresholds to discriminate patients requiring transfusion (≥ 3 PRBCs vs < 3 PRBCs) were: fibrinogen blood concentration < 1.5 g/L, EXTEM CFT > 159 s, INTEM CFT > 110 s and FIBTEM MCF < 9 mm.

**Table 4:** Prediction of packed red blood cells transfusion (at least 3 units) based on standard laboratory tests and ROTEM tests

Coagulation test	Threshold	Sensitivity TPR (%)	Specificity TNR (%)	PPV (%)	NPV (%)	FPR (%)	FNR (%)	Correctly classified (%)
Conventional coagulation tests								
Quick, %	< 70	35.1	86.8	65.0	65.7	13.2	64.9	65.6
INR, a.u.*	> 1.3	21.9	97.5	87.5	60.9	2.5	78.1	63.9
PTT, s	> 35	30.6	85.7	61.1	62.7	14.3	69.4	62.4
Fibrinogen, g/L	< 1.5	25.6	100	100	64.6	0.0	74.4	68.5
Platelets, 10^3^/mcL	< 100	26.3	82.0	52.6	59.4	18.0	73.7	58.0
Coagulopathy#, %		59.4	66.7	48.7	75.5	33.3	40.6	64.1
Rotational Thromboelastometry								
Extem								
CT (s)	> 80	30.8	90.6	70.6	64.0	9.4	69.2	65.2
CFT (s)	> 159	40.5	88.2	71.4	67.2	11.8	59.5	68.2
α Angle	< 63	30.4	96.4	87.5	62.8	3.6	69.6	66.7
CA15 (mm)	< 45	35.9	92.5	77.8	66.2	7.5	64.1	68.5
MCF (mm)	< 50	38.2	85.1	65.0	65.6	14.9	61.8	65.4
Intem								
CT (s)	> 240	2.6	98.1	50.0	57.8	1.9	97.4	57.6
CFT (s)	> 110	41.0	90.0	76.2	66.2	10.0	59.0	68.5
α Angle	< 70	32.0	86.2	66.7	59.2	13.8	68.0	61.1
CA15 (mm)	< 45	23.1	94.0	75.0	61.0	6.0	76.9	62.9
MCF (mm)	< 50	17.9	98.0	87.5	60.5	2.0	82.1	62.9
Fibtem								
CA15 (mm)	< 8	30.8	96.2	85.7	64.9	3.8	69.2	68.1
Fibtem MCF (mm)	< 9	33.3	96.2	86.7	66.2	3.8	66.7	69.6
Aptem								
CT (s)	> 80	16.0	96.4	80.0	56.3	3.6	84.0	58.5
CFT (s)	> 159	32.0	89.3	72.7	59.5	10.7	68.0	62.3
α Angle	< 63	28.0	92.9	77.8	59.1	7.1	72.0	62.3
CA15 (mm)	< 45	39.1	89.3	75.0	64.1	10.7	60.9	66.7
MCF (mm)	< 50	28.0	90.3	70.0	60.9	9.7	72.0	62.4
Coagulopathy#, %		78.9	67.1	38.5	92.5	32.9	21.6	69.6

*TPR* true positive rate, *TNR* true negative rate, *PPV* positive predictive value, *NPV* negative predictive value, *FPR* false positive rate, *FNR* false negative rate, *INR* international normalized ratio, *a.u.* arbitrary unit, *PTT* partial thromboplastine time, *CT* coagulation time, *CFT* clot formation time, *INR* international normalized ratio, *MCF* maximal clot firmness

#Coagulopathy

• Using standard coagulation tests, coagulopathy was defined as one or more of the following results: Quick <70%, INR > 1.3, fibrinogen <1.5 g/L, aPTT >35 s, and platelet count <100,000/mcL

• Using ROTEM results, coagulopathy was defined as two or more of the following results: EXTEM CT > 80 s, EXTEM CFT > 159 s, EXTEM MCF < 50 mm, INTEM CT > 240 s, INTEM CFT > 110 s, INTEM MCF < 50 mm, and FIBTEM MCF < 9 mm

Coagulation parameters of SCTs and ROTEM analysis all normalized 18–24 h after surgery (Table 5), except the platelet count that remained decreased by 28% (IQ25–75, 12–41%) compared with preoperative values and, it was lower in the HB than in the LB group. Postoperative clinical outcome did not differ significantly between the two groups, pneumonia (24%) and bleeding (10%) being the most frequent complications.

**Table 5:** Postoperative coagulation data and clinical outcome in patients undergoing emergent neurosurgery

Outcome	High Bleeders	Low Bleeders	*P*-value
	(N = 39)	(N = 53)	
Coagulation testing one day after surgery			
Platelets, 10^3^/ml*	109 (38)	159 (65)	0.002†
Quick, %^a^	90 (72–99)	92 (75–100)	0.611^b^
INR, a.u*	1.11 (0.13)	1.12 (0.12)	0.578†
Fibrinogen, g/L*	4.0 (1.2)	3.9 (1.2)	0.326†
EXTEM CT	57 (42–72)	55 (40–70)	0.641
EXTEM MCF	62 (50–74)	59 (48–71)	0.284
INTEM MCF	66 (59–75)	62 (56–71)	0.248
FIBTEM MCF	58 (51–65)	57 (50–64)	0.968
Clinical Outcome (in-hospital)			
Composite Mortality - Morbidity	47 (88.7)	15 38.5	0.001
Mortality	14 (35.9)	11 (20.8)	0.107
Cardiovascular outcomes			
Myocardial infarction/ischemia	1 (2.6)	0 (0.0)	0.424^c^
Cardiac arrhythmia	3 (7.7)	5 (9.4)	1.000^c^
Thromboembolic complication	1 (2.6)	2 (3.8)	1.000^c^
Acute heart failure	1 (2.6)	2 (3.8)	1.000^c^
Respiratory complications			
Pneumonia	8 (20.5)	14 (26.4)	0.424
Acute Respiratory Distress Syndrome	0 (0.0)	1 (1.9)	1.000^c^
Mechanical ventilation, days^a, d^	3 (1–11)	3 (1–13)	0.673^b^
Renal dysfunction			
Reduction in GFR > 25%	6 (15.4)	3 (5.7)	0.161^c^
Other complications			
Infection	3 (7.7)	5 (9.4)	1.000^c^
Postoperative bleeding	6 (15.4)	7 (13.2)	0.767
Length of stay			
ICU, days^a, d^	10 (3–19)	5.5 (3–16)	0.391^b^
Hospital, days^a, d^	31 (18–37)	23.5 (15–32)	0.173^b^

Data given as number (percentage) unless otherwise indicated. *GFR* glomerular filtration rate, *ICU* intensive care unit

Chi-squared tests were used for statistical tests unless otherwise indicated;

^a^Data given as median (range)

^b^Wilcoxon rank sum test

^c^Fisher exact test

^d^Survivors only

*mean (standard deviation)

+unpaired Student t test

## Discussion

This cohort study provides for the first time some insight into the coagulation pattern of patients undergoing emergent neurosurgical interventions. Our data showed that: (i) a coagulopathic pattern was detected preoperatively in 35% patients based on SCTs and in 21% based on ROTEM analysis; (ii) the results obtained with ROTEM fairly correlated with SCTs; (iii) ROTEM analysis had a higher sensitivity to predict the need for transfusion of at least 3 PRBC than SCTs; (iv) abnormalities in ROTEM parameters were useful to guide the correction of hemostatic abnormalities.

In elective neurosurgery, preexisting coagulation disorders are rarely diagnosed (less than 3%) and the sensitivity of any SCTs in predicting clinical outcome, particularly the need for homologous transfusion, is less than 10% [23, 24]. Before emergent neurosurgical interventions, these SCTs are time consuming and their diagnostic utility is limited to the initial formation of fibrin strands [11]. In contrast, POC-VHAs such as ROTEM provide on-line information from the initiation to full development of blood clot including subsequent fibrinolysis and this emerging testing modality have proven its superiority in managing acutely bleeding patients compared with SCTs [25]. As expected, we found a good correlation between PTT and intrinsically activated ROTEM tests (INTEM CT and alpha angle) at the early stage of coagulation whereas plasma fibrinogen levels correlated fairly well with the clot amplitude of the FIBTEM test (MCF and CA15). In elective surgical pediatric cases, Haas et al. reported a strong correlation between fibrinogen plasma levels and FIBTEM CA (*R* = 0.88) and between PT and INTEM CT [26]. Likewise, in ninety patients admitted with variable degree of trauma, Rugeri et al. also observed a good correlation between SCTs and ROTEM parameters, namely between PT and EXTEM CA as well as between fibrinogen levels and FIBTEM CA [27].

In the present study, 32 out of 92 patients presented prolonged PT/PTT, low levels of fibrinogen and/ or low platelet counts that have been used as criteria to define an acute coagulopathy. With the POC-VHA, abnormalities in coagulation profile were reported in 19 patients based on ROTEM parameters using validated cut-off values recommended by the manufacturer [18] and, agreement between SCTs and ROTEM was achieved in 66.3% for the diagnosis of trauma-induced coagulopathy. In a recent multicenter trial including trauma patients, Hagemo et al. confirmed that clot amplitude with ROTEM analysis was a valid marker of an ongoing coagulopathy, with optimal thresholds of FIBTEM CA < 9 mm and EXTEM CA5 ≤ 37 mm [28].

Since small intracranial hematomas or ongoing bleeding may have devastating effects, transfusion of at least 3 PRBCs was used as a surrogate to define major bleeding among our neurosurgical patients [29–31]. This contrasts with cardiac surgery, liver transplantation and multiple trauma where cut-off values for major bleeding are ranging from 4 to 10 PRBCs and are associated with a high burden of morbidity and mortality [32, 33]. Not surprinsingly, the largest AUC (0.74) were obtained in patients with the lowest Glasgow Coma Score that reflected the severity of the initial brain injuries and the consequent trauma-induced coagulopathy with alteration in POC-VHA and SCTs. Our data demonstrated that the amplitude of clot formation at the FIBTEM and EXTEM assays were the most accurate ROTEM-derived parameters in predicting transfusion requirement and were at least equivalent to the results obtained with SCTs using well-established thresholds. Interestingly, preoperative treatment with anticoagulants and antiplatelets did not alter the predictive value of ROTEM-derived parameters for transfusion requirement. This could be explained by the fact that, clinicians, − being aware of these drug-induced hemostatic effects -, administered FFP and/or thrombopheresis to minimize intraoperative bleeding.

These findings are consistent with studies involving patients with multiple trauma, although different thresholds of clot amplitude have been identified with platelet-inhibited and tissue factor-activated assays (FIBTEM and EXTEM). Davenport reported that, EXTEM CA5 ≤ 35 mm was a better predictor of massive transfusion (≥ 10 PRBCs) than INR > 1.2 (71% vs 43%) [19]. In other cohort studies, a satisfactory detection rate for massive transfusion was reported using thresholds of fibrinogen ≤1.9 g/L, FIBTEM CA5 < 9 mm and FIBTEM MCF < 7 mm [28, 34].

Besides early identification of coagulation deficits, there is now growing evidence that POC-VHA coupled with determination of blood hemoglobin concentration facilitates goal-directed blood management and hemostatic therapy, particularly in cardiac surgery and in massive trauma [35, 36]. In a randomized controlled trial including 100 high-risk cardiac surgical patients, Weber et al. demonstrated that hemostatic therapy based on viscoelastic and aggregometric testing was associated with reduced exposure to allogenic blood products, faster weaning from the ventilator and earlier discharge from the ICU, compared with empiric hemostatic therapy based on SCTs and clinical judgement [37]. In the present study, we implemented a simple algorithm based on perioperative ROTEM analysis to correct the early coagulation disorders. Accordingly, the administration of platelet concentrates, PCC, FFP, fibrinogen and tranexamic acid was guided using specific thresholds that have been previously reported [23, 27–29]. This individualized goal-directed hemostatic approach resulted in normalizing both ROTEM-derived parameters and SCTs on the first day after surgery, except for the platelet count. Considering the multifactorial pathophysiology of perioperative hemorrhage during neurosurgery, SCTs are of limited diagnostic value and have a long turnaround time exceeding 60 min [38]. In contrast, POC-VHA results are rapidly available and, by analyzing the interactions between plasma coagulation factors and circulating blood cells, they provide valuable information regarding the causes of perioperative hemorrhage [39].

This prospective study has several limitations. First, this was a single center study with a relatively small and heterogenous sample of patients with different acute brain disorders, comorbidities and treatments. Second, the different timings of blood sampling for coagulation testing could likely influence the results but reflected the practical aspects of managing these critically-ill surgical patients. Indeed, blood for ROTEM analysis was collected later than for SCTs, −after the initiation of resuscitative/stabilization interventions and closer to the start of surgery-, therefore ROTEM parameters might better characterize the ongoing coagulation abnormalities. Third, a quarter of patients were on preoperative antiplatelet or anticoagulant therapy and, ROTEM analysis was poorly suited to detect any impairement in platelet adhesion/agregation and in vitamine K-dependent coagulation factor formation that could explain the observed poor correlation between INR/Quick and EXTEM CT. Finally, hyperfibrinolysis could not be detected in this cohort since tranexamic acid was administered prophylactically in 18% of HB patients, namely those with severe brain trauma.

## Conclusions

Implementation of POC-VHAs provided useful indicators for detecting and correcting hemostatic abnormalities in patients undergoing emergent neurosurgery. ROTEM assays were equal or superior to SCTs in identifying patients with acute coagulopathy and in predicting intraoperative blood transfusion requirement. Future studies should be directed to evaluate the clinical impact of repeated POC-VHA measurements in guiding haemostatic treatment in emergent neurosurgery.

## Abbreviations

AIS: Abbreviated Injury Scale
AUC: aera under the curve
CA15: clot amplitude after 15 min
CFT: clot firmess time
CT: coagulation time
FFP: fresh frozen plasma
HB: high bleeder
ICU: intensive care unit
INR: international normalized ratio
LB: low bleeder
MCF: maximum clot firmness
POC: Point-Of-Care
PPC: prothrombin complex concentrate
PRBC: packed red blood cell
PT: prothrombin time
PTT: partial thromboplastine time
rFVII: recombinant factor
ROTEM: Rotational elastometry
SCT: standard coagulation test
TA: tranexamic acid
TEG: thromboelastography
VHA: viscoelastic hemostatic assay
